# Naphtha (petroleum), hydrotreated heavy: MAK Value Documentation, addendum – Translation of the German version from 2025

**DOI:** 10.34865/mb6474248yole11_1ad

**Published:** 2026-03-30

**Authors:** Andrea Hartwig, MAK Commission

**Affiliations:** 1 Institute of Applied Biosciences, Department of Food Chemistry and Toxicology, Karlsruhe Institute of Technology (KIT) Adenauerring 20a, Building 50.41 76131 Karlsruhe Germany; 2 Permanent Senate Commission for the Investigation of Health Hazards of Chemical Compounds in the Work Area, Deutsche Forschungsgemeinschaft, Kennedyallee 40, 53175 Bonn, Germany. Further information: Permanent Senate Commission for the Investigation of Health Hazards of Chemical Compounds in the Work Area | DFG

**Keywords:** naphtha (petroleum), hydrotreated heavy, central nervous system, neurotoxicity, MAK value, maximum workplace concentration, peak limitation, Luft, air

## Abstract

The German Senate Commission for the Investigation of Health Hazards of Chemical Compounds in the Work Area (MAK Commission) summarized and re-evaluated the data for the derivation of the occupational exposure limit value (maximum concentration at the workplace, MAK value) for naphtha (petroleum), hydrotreated heavy [64742-48-9]. Relevant studies were identified from a literature search and also unpublished study reports were used. The critical effect is preclinical neurotoxicity. The results of a 13-week study with an isoparaffinic product in rats and a behavioural toxicity study in volunteers do not contradict the previous MAK value of 50 ml/m^3^. The assignment to Peak Limitation Category II with an excursion factor of 2 is retained. Additionally, there are no experimental data showing that juvenile animals are more sensitive to naphtha-induced neurotoxic effects than adults. Therefore, there is no evidence that would require changing the previous categorization from Pregnancy Risk Group D to Pregnancy Risk Group B (suspected).

**Table:** 

**MAK value (2009)**	**50 ml/m^3^ (ppm) ≙ 300 mg/m^3^**
**Peak limitation (2009)**	**Category II, excursion factor 2**
	
**Absorption through the skin**	**–**
**Sensitization**	**–**
**Carcinogenicity**	**–**
**Prenatal toxicity (2009)**	**Pregnancy Risk Group D**
**Germ cell mutagenicity**	**–**
	
**BAT value**	**–**
	
Synonyms	hydrocarbons, paraffinic and naphthenic, C_6_–C_13_, boiling points between 65 and 230 °C, aromatic-free Stoddard Solvent IIC^a)^ white spirit, dearomatized (white spirit type 3)^a)^
CAS number	64742-48-9
**1 ml/m^3^ (ppm) ≙ 6 mg/m^3^**	**1 mg/m^3^ ≙ 0.167 ml/m^3^ (ppm)**

^a)^ contains only higher-boiling components

This addendum re-evaluates the MAK value, among other things, as 2 studies in humans and animals were conducted that were not available for the 2009 documentation (Hartwig 2015) but are described in the addendum to “Distillates (petroleum), hydrotreated light” (CAS No. 64742-47-8; Hartwig and MAK Commission 2017). These are relevant also for hydrotreated heavy naphtha (petroleum).

White spirit type 3 (dearomatized “white spirit”) corresponds to CAS number 64742-48-9, but is only a partial equivalent, as the main components of white spirit type 3 are C_9_–C_11_ hydrocarbons.

The data in the ECHA registration dossier (ECHA 2023) for CAS number 64742-48-9, on the other hand, are based almost exclusively on studies with unleaded petrol (CAS number 86290-81-5 and CAS number 8006-61-9) with a boiling range of 30 to 260 °C.

## Effects in Humans

The NOAEC (no observed adverse effect concentration) for the exposure of test persons (Lammers et al. 2007 in Hartwig 2015) to aromatic “white spirit” for 4 hours is about 600 mg/m^3^ (100 ml/m^3^). Neuropsychological tests did not reveal any significant impairments or local effects at this concentration. After exposure to an aromatic mixture for 30 minutes, the NOAEC for effects on the central nervous system (CNS) measured in performance tests was 400 ml/m^3^ and the LOAEC (lowest observed adverse effect concentration) was 680 ml/m^3^ (Gamberale et al. 1975 in Hartwig 2015).

In a volunteer study, no significant behavioural toxicity was observed after exposure for 4 hours to low aromatic white spirit and aromatic white spirit up to the highest concentration tested of 300 mg/m^3^ (50 ml/m^3^) (Hartwig and MAK Commission 2017; Juran et al. 2014).

## Animal Experiments and in vitro Studies

In a study from 1980 with an aromatic-free product (C_10_–C_12_ isoparaffins, 170 °C to 187 °C), groups of 18 male and 18 female Wistar rats (10 to 13 weeks old) were exposed to concentrations of 0, 359, 737 or 1444 ml/m^3^ in whole-animal exposure chambers on 5 days a week, for 6 hours a day, for 13 weeks (Carrillo et al. 2013; Shell 1980). The detailed description of the study and the evaluation of the results are taken from Hartwig and MAK Commission (2017) below:

“The anaemia was only very mild, there was no clear dependence on the concentration and it occurred only in the males. However, mild anaemia in male rats was found also after exposure to other aliphatic solvents (Carrillo et al. 2013). The authors gave the normal variations in blood parameters as a possible explanation. They pointed out that these changes were consistent with those described as anaemia of chronic disease. Such changes were often observed in studies with high doses and are not toxicologically relevant (Car et al. 2006). At 1100 mg/m^3^ and above, the study using Stoddard Solvent IIC (C_10_–C_14_, level of aromatics: maximum of 1%; NTP 2004) revealed mild anaemia in male F344 rats after 3 months, but not in females or in male and female B6C3F1 mice. Stoddard Solvent IIC also caused alpha2u nephropathy only in male F344 rats. Therefore, anaemia is assumed to be a secondary effect. The NTP considered the findings of anaemia to be toxicologically irrelevant (NTP 2004). In the high concentration group, reduced leukocyte counts were observed only in male rats. Leukocyte numbers vary considerably among rat strains (Car et al. 2006). Furthermore, the initial age of the animals is of importance for evaluating this effect because leukocyte counts decrease in the course of a rat’s life (NTP 2004). Most other aliphatic solvent mixtures had no effects on the leukocyte count. Lethargy occurred in the high concentration group after exposure. This is an adverse effect on the CNS. The authors considered the increased liver weights to be an adaptive effect because no histological findings were obtained in the liver and ASAT and ALAT activities were not increased. This effect may have been caused by the induction of xenobiotic-metabolizing enzymes, but this was not investigated. The Commission considers a 40% increase in the absolute liver weights of the high concentration group by enzyme induction to be adverse because it may interfere with metabolic processes. However, the middle concentration that induced a 14% (incorrect in the quoted text: 13%) increase may be regarded as the NOAEC. There was a concentration-dependent increase in the incidences of inflammatory infiltrates in the lungs of the males. The authors stated that this finding was often observed in the rats from the test laboratory and that the incidence was just as high in the control animals. However, no data were reported. The absolute kidney weights of the female rats were increased in the high concentration group without histological findings. The kidney findings obtained in males were caused by alpha2u nephrotoxicity; they are thus not relevant to humans. In the high concentration group, various effects were found that the Commission regarded as substance-induced and adverse; therefore, 1444 ml/m^3^ is regarded as the LOAEC (lowest observed adverse effect concentration) and 737 ml/m^3^ (incorrect in the quoted text: 759 ml/m^3^) as the NOAEC.”

## Manifesto (MAK value/classification)

The critical effect of hydrotreated heavy naphtha (petroleum) is its effect on the central nervous system (Hartwig 2015).

**MAK value. **In a 13-week inhalation study carried out in Wistar rats with a product free of aromatics (C_10_–C_12_ iso­paraffins, 170 °C to 187 °C), increased liver weights were found as the most sensitive end point at the lowest concentration of 359 ml/m^3^ and above, but no histopathological or clinico-chemical (ASAT/ALAT) effects were detected. The liver weights were increased by less than 10% at 359 ml/m^3^, by 14% at 737 ml/m^3^ and by about 40% at 1444 ml/m^3^. After exposure to the high concentration, CNS effects in the form of lethargy were found (Carrillo et al. 2013; Shell 1980). The systemic NOAEC was 737 ml/m^3^. Human studies have yielded significantly lower NOAEC and LOAEC values, which is why the MAK value is derived from human studies rather than animal studies:

Based on acute behavioural toxicity studies in humans (4-hour NOAEC 100 ml/m^3^; 30-minute NOAEC 400 ml/m^3^, LOAEC 680 ml/m^3^), a MAK value of 50 ml/m^3^ was established for hydrotreated heavy naphtha (petroleum) (Hartwig 2015). The MAK value takes into account also the increased respiratory volume at the workplace and accumulation over the working week (Hartwig and MAK Commission 2017). The new human study of Juran et al. (2014) with a 4-hour NOAEC of 50 ml/m^3^ does not contradict this, as higher concentrations were not tested. The MAK value has therefore been retained. The boiling range of 65 to 230 °C is lower than that of hydrotreated light distillates (petroleum) (150–290 °C). Possible aerosol formation is thus less likely and, unlike in the case of hydrotreated light distillates (petroleum), a MAK value has not been set for the respirable aerosol fraction of hydrotreated heavy naphtha (petroleum).

**Peak limitation. **Due to its systemic effects, hydrotreated heavy naphtha (petroleum) remains assigned to Peak Limitation Category II.

In view of the calculated initial half-life in the brain of less than 2 hours (Hartwig 2015), acute CNS effects are expected to set in and subside rapidly, which is why an excursion factor of 2 has been set. The resulting permissible short-term concentration of 100 ml/m^3^ has been shown not to cause local irritation in test persons and is below the 30-minute NOAEC of 400 ml/m^3^ (see “MAK value”).

**Prenatal toxicity. **The MAK value for hydrotreated heavy naphtha (petroleum) of 50 ml/m^3^ (300 mg/m^3^) has been re-evaluated and confirmed. The critical effects for the derivation of the MAK value are local effects and neurotoxicity. Prenatal toxicity studies in rats did not reveal any effects up to 900 ml/m^3^ (5000 mg/m^3^) (C_9_–C_13_ mixture). In 2009, hydrotreated heavy naphtha (petroleum) was assigned to Pregnancy Risk Group D because no valid data for developmental neurotoxicity were available (Hartwig 2015). There are no experimental data showing that juvenile animals are more sensitive to neurotoxic effects induced by hydrotreated heavy naphtha (petroleum) than adults. Therefore, there is no evidence that would require changing the previous categorization from Pregnancy Risk Group D to Pregnancy Risk Group B (suspected).

**Carcinogenicity. **There are no new studies relevant to the assessment. The substance has therefore not been classified in one of the categories for carcinogens (see Hartwig 2015).

**Germ cell mutagenicity. **There are no new studies relevant to the assessment. The substance has therefore not been classified in one of the categories for germ cell mutagens (see Hartwig 2015).

**Absorption through the skin. **The substance is not designated with an “H” based on an in vitro study with rat skin (see Hartwig 2015). A review confirms that dermal exposure to petroleum substances is low and does not pose a health risk under the usual conditions of use (Jakasa et al. 2015). The substance has therefore not been designated with an “H” (for substances which can be absorbed through the skin in toxicologically relevant amounts).

**Sensitization. **There are no data available for the skin sensitizing potential of the substance in humans. Furthermore, no new animal studies are available or data from alternative methods without animal testing. There are no data available for the sensitizing potential on the airways. The substance has therefore not been designated with “Sh” or “Sa” (for substances which cause sensitization of the skin or airways).
